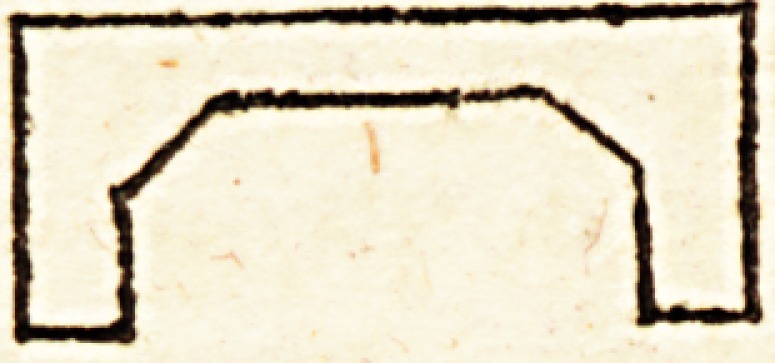# Mr. Harrold's Machine for Fractured Thighs

**Published:** 1806-04

**Authors:** E. Harrold

**Affiliations:** Cheshunt, Herts


### *To the Editors of the Medical and Physical Journal.*

GENTLEMEN,

YOU will oblige me by the insertion of the following additional remarks in your next Number, on the machine for fractures of the thigh, of which an account was given in your eighty-third Number.

As there may be some danger, when a patient is moved to any distance upon the machine, of the lower ends of the bolts (B. B. fig. 2.) getting out of the notches upon the main main frame at (R. fig. 1.) by any sudden jerk of the bearers or involuntary motion of the patient; that danger may be obviated by a ring being screwed into the upper side of the lower end of each bolt, through which a strap may be passed, and buckled securely round the frame.

The screw (U. fig. 1 and fig. 3.) should be set more obliquely upwards than represented (in fig. 1.) in order that when the extremity (W. fig. 3.) is elevated to the utmost, the knobbed extremity of the screw U. may be at the same distance from the hinge at V. that it was at its entrance at U. The screw U. ends in a knob with a short neck, by which it is fastened through a perforation in a small iron plate to (V. W. fig. 3.)

If the lower buckles (I. I. fig. 1.) of the leg splint are fixed so as not to have any lateral motion, it will be necessary for the lower staple (b) near the footboard (X. fig. 3.) to be two inches wide, as the distance from (A. to W. fig. 3.) will be lessened by the elevation of W.

The footboard iron (fig. 4.) not being quite correctly represented, requires a little explanation. It is a square iron frame of this form:

The spaces between A B and A B are equal to the thickness of the end of the support, (W fig 3), the internal projecting piece A A being designed to slide upon the edges of the support where the hollow is made for the reception of the heel; and the spaces between A C and A C are designed for the cushion of wool, which forms the bed for the limb. To prevent any chance of it sliding down or being lost, a short thick screw might be made at D, to press against the bottom of the support; but that cannot happen from the pressure of the patient's foot. The holes at C C are for the screws which form the pivot of the board, and its elevation is regulated by the screw E. The neck through which this screw passes, and the upper part of the frame from C to C, should be made tolerably strong to resist the pressure of the foot against the board. In cases of compound fracture, or of wounds in addition to fracture, the support (fig. 3) will be liable to be disagreeably bly stained. To prevent this it may be made of lime-tree wood, which will take a brown stain with oil.

My second sized support (fig. 3) measures from D to A, 11 inches; from A to V, 5 inches; from V to W, 12½ inches. Its greatest width 5 inches, and its least 4⅛ inches, with splints of a corresponding size. This will suit men under the middle size, and women generally.

My smallest sized support (fig. 3) measures from D to A, 8¼ inches; from A to V, 3¾ inches; from V to W 10½ inches; its greatest width 4⅜ inches; its least 3½ inches. This will suit children from six or seven years to twelve or fourteen; the hollow for the reception of the heel in all the supports should be made about six inches long, to suit legs of different lengths, and that the longest admissible length of the shorter size may be equal to the shortest admissible length of the size next above it. I have not given the above proportions as the best, but as a guide to those gentlemen who may be disposed to construct a similar instrument, until further experience points out a better. It will be useful to have the lower strap of the leg splint (I fig. 1) as distant from the lower end of the splint as it conveniently may, that it may not be in the way of the foot where the support is made for the shortest leg that it will admit. As the elevation of (W. fig. 3) shortens the distance from A to W, and as this elevation will always be necessary in a muscular leg, it follows, that a short *muscular* leg will reach the hollow for the heel when a short leg that is *not muscular* will not. It will save the surgeon some trouble if the notches upon the main frame at R are numbered, by figures painted on the side, or written upon paper with sufficient accuracy, and then pasted on each side of the frame. The notches of the bolts QQ (fig. 2) are prevented from taking hold of the *lower* staple, by the angles of the bolts being filed off, and by the lower staple being made of this form:

The *upper* staple has its upper edge made thinner than its under edge, by which means it is accommodated to the notches in the bolt.

I am, &c.

*Cheshunt, Herts, March* 5, 1806. E. HARROLD.

## Figures and Tables

**Figure f1:**
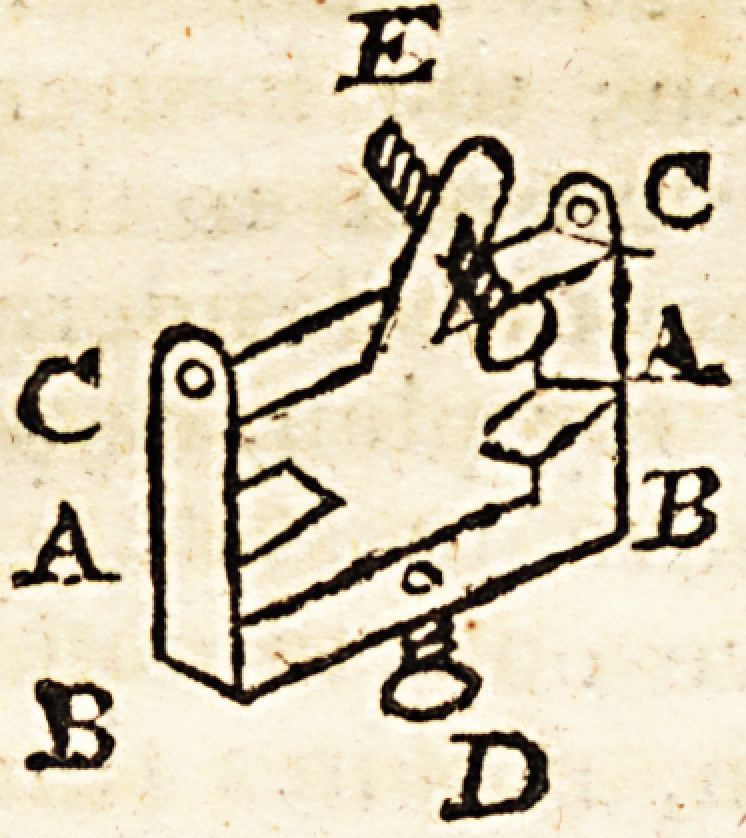


**Figure f2:**